# Antibiofilm Activity of a *Trichoderma* Metabolite against *Xanthomonas campestris* pv. *campestris*, Alone and in Association with a Phage

**DOI:** 10.3390/microorganisms8050620

**Published:** 2020-04-25

**Authors:** Marina Papaianni, Annarita Ricciardelli, Andrea Fulgione, Giada d’Errico, Astolfo Zoina, Matteo Lorito, Sheridan L. Woo, Francesco Vinale, Rosanna Capparelli

**Affiliations:** 1 Department of Agricultural Sciences, University of Naples Federico II, 80055 Portici (NA), Italy; marina.papaianni@unina.it (M.P.); zoina@unina.it (A.Z.); lorito@unina.it (M.L.); capparel@unina.it (R.C.); 2 Department of Chemical Sciences, University of Naples Federico II, 80125 Naples, Italy; ananrita.ricciardelli@unina.it; 3 Istituto Zooprofilattico Sperimentale del Mezzogiorno (IZSM), 80055 Portici (NA), Italy; Andrea.Fulgione@izsm.it; 4 Institute for Sustainable Plant Protection, National Research Council, 80055 Portici (NA), Italy; woo@unina.it; 5 Department of Pharmacy, University of Naples Federico II, 80131 Naples, Italy; 6 Department of Veterinary Medicine and Animal Productions, University of Naples Federico II, 80137 Naples, Italy

**Keywords:** *Xanthomonas campestris* pv. *campestris*, 6-pentyl-α-pyrone, antibiosis, *Trichoderma*, secondary metabolites, antibiofilm, Gram-negative bacterium, antibiotic resistance

## Abstract

Biofilm protects bacteria against the host’s immune system and adverse environmental conditions. Several studies highlight the efficacy of lytic phages in the prevention and eradication of bacterial biofilms. In this study, the lytic activity of Xccφ1 (*Xanthomonas campestris* pv. *campestris*-specific phage) was evaluated in combination with 6-pentyl-α-pyrone (a secondary metabolite produced by *Trichoderma atroviride* P1) and the mineral hydroxyapatite. Then, the antibiofilm activity of this interaction, called a φHA6PP complex, was investigated using confocal laser microscopy under static and dynamic conditions. Additionally, the mechanism used by the complex to modulate the genes (*rpf, gumB, clp* and *manA*) involved in the biofilm formation and stability was also studied. Our results demonstrated that Xccφ1, alone or in combination with 6PP and HA, interfered with the gene pathways involved in the formation of biofilm. This approach can be used as a model for other biofilm-producing bacteria.

## 1. Introduction

The Gram-negative bacterium *Xanthomonas campestris* pv. *campestris* (*Xcc*) is the causal agent of black-rot disease in crucifers, responsible for serious yield losses worldwide [1]. The bacterial secretion composed of exopolysaccharides can obstruct the xylem vessels, causing tissue necrosis and leaf wilting [2]. The *Xcc* produces a variety of substances, such as enzymes, that may be used by this bacterium to parasitize the host [3].

At present, the ongoing spread of antibiotic-resistant bacteria is a major public health concern that is further exacerbated by some agricultural management practices [4]. The bacterial contamination of food surfaces is the main cause of food-borne illnesses [5]. The mechanism mainly used by bacteria to improve its chance of survival, especially in weakly resistant isolates, is the formation of biofilm [6], an extracellular polymeric matrix composed of proteins, polysaccharides and DNA [7]. In this context, emerging issues in the management of infections caused by antibiotic-resistant and biofilm-forming bacteria have encouraged the development of alternative therapeutic approaches [8,9].

Several studies highlight the efficacy of lytic phages against bacteria [10,11]; however, antiphage resistance mechanisms may limit their activity [12]. We believe that this strategy, combined with the application of metabolites obtained from a specific (or given) fungus, might be a potential alternative in the prevention and control of biofilm-related infections. Thus, *Trichoderma,* a well-known beneficial fungus, was selected for its plant growth promotion [13] and biocontrol abilities [14]. The species *T. atroviride* produces a natural compound, known as 6-pentyl-α-pyrone (6PP), with interesting properties (i.e., antibiotic, plant metabolome interference) [13,15].

Interactions between biomolecules and different kinds of inorganic surfaces, like hydroxyapatite (HA) nanocrystals, biogenic silica, carbonates and phosphates, are important in numerous biological applications, such as drug and gene delivery, a possible way to selectively release prodrug in tumor tissues, and antimicrobial molecule carriers [16,17,18]. Previous studies have demonstrated that HA nanocrystals, a mineral rich in Ca_10_(PO_4_)_6_(OH)_2_, can be used for bacteriophage delivery and as an enhancer of its biological activities and stability [16]. Therefore, HA may act as carrier of antimicrobial compounds [17] and as a factor to improve lytic activity of bacteriophages [16], adsorbing molecules as well as particles [18].

This is the first study to describe the antibiofilm activity of 6PP, alone and in association with a phage and hydroxyapatite (a biocompatible mineral) against *Xcc.* This association may overcome the problems arising from the formation of bacterial biofilm during infection. In particular, the antibiofilm activity of 6PP helps the phage to better interact with and lyse the bacteria, and the HA can be considered as a potential carrier for the active principles.

## 2. Materials and Methods

### 2.1. Isolation and Growth of Xcc Phages

Ten grams of rhizospheric soil collected from different kohlrabi (*Brassica oleracea* var. *gongylodes*) plants showing black rot symptoms was suspended in 15 mL of nutrient broth (Sigma Aldrich) and agitated for 30 min at 20 °C. Soil sediment was removed by centrifugation (5.000 rpm for 20 min), and the supernatants were transferred to sterile flasks. *Xcc* was incubated overnight at 24 °C under shaking conditions. Cultures were centrifuged and filtered through a 0.22 μm pore-size membrane filter (MF-Millipore, Darmstadt, Germany). The filtrates were assayed for the presence of *Xcc*-infecting phage by plating a 10 μL aliquot on soft agar. After 48 h, plaque-forming units (PFU) of phage were picked and resuspended in distilled water, incubated at 37 °C for 4 h, centrifuged at 5000 rpm for 30 min and filtered as mentioned above [19]. The experiment was repeated five times.

### 2.2. Complex φHA6PP

The φHA6PP complex was prepared by mixing 1 mL of HA (100 mg/mL) with 1 mL of Xccφ1 (10^8^ PFU/mL) and varying concentrations of 6PP (0.02, 0.01, 0.004, 0.002, 0.001, 0.0004 and 0.0002 μg/mL solubilized in dimethyl sulfoxide (DMSO)). A control sample with the solvents alone was also prepared. The mixture was incubated at room temperature, under shaking conditions, for time periods of 0, 30, 90, 180 and 300 min and 24 h. Later, samples were centrifuged, and the pellet was suspended in distilled water. A further centrifugation step was carried out, and the supernatant was screened for the presence and concentration of phage particles using the double-layer agar (DLA) method [16]. After overnight incubation at 24 °C, the optimal incubation time was selected.

### 2.3. Isolation of 6-Pentyl-α-pyrone

The biocontrol fungus *T. atroviride* strain P1 was maintained on potato dextrose agar (PDA, Sigma St. Louis, MO, USA) Petri plates at room temperature and sub-cultured bimonthly. Six 5 mm diameter plugs were collected from the actively growing margins of the PDA cultures and inoculated into 5 L conical flasks containing 1 L of sterile potato dextrose broth (PDB, Sigma). The stationary cultures were incubated at 25 °C for 21 days. The cultures were vacuum filtered through filter paper (Whatman No. 4; Brentford, UK). The culture broth of *T. atroviride* strain P1 was extracted exhaustively with ethyl acetate (EtOAc). The red-brown residue was subjected to flash column chromatography (Si gel; 50 g) by eluting with a gradient of EtOAc:Petroleum ether (8:2 to 10:0). Analytical thin-layer chromatography (TLC) was performed, and fractions were further purified by using silica-gel flash chromatography (Kieselgel 60, GF_254_, 0.25 and 0.5 mm, Merck Darmstadt, Germany). Compounds were detected with UV radiation (254 or 366 nm) and by dipping the TLC plates in a 10% (*w*/*v*) aqueous solution of CeSO_4_ or in a 5% (*v*/*v*) ethanol solution of H_2_SO_4_ and heating at 110 °C for 10 min. Pure metabolite was characterized by LC/MS q-TOF analysis recorded with an Agilent system (HPLC 1260 Infinity Series) coupled to a q-TOF mass spectrometer Model G6540B with a dual electrospray ionization source and equipped with a DAD system (Agilent Technologies, Santa Clara, CA, USA). This compound showed the same spectrometric data of an authentic standard previously isolated in our laboratories [15].

### 2.4. Antibiofilm Activity of 6-Pentyl-α-pyrone

The biofilm formation was measured using crystal violet staining. The experiment was performed to characterize the antibiofilm activity of different concentrations of 6PP on mature biofilm. A 200 μL of *Xcc* was added to each well using a 96-well plate (Falcon), and incubated at 24 °C for 72 h under static conditions to allow bacterial attachment and the biofilm formation. Then, 6PP was added at different concentrations to the complex. After 4 h, the samples were analyzed as previously reported [20].

### 2.5. CLSM Analysis for Static Biofilm Evaluation

Confocal laser scanning microscopy (CLSM) of *Xcc* biofilms was performed on Nunc Lab-Tek eight-well Chamber Slides (No. 177445; Thermo Scientific, Ottawa, ON, Canada). The overnight cultures of *Xcc* were diluted to a cell concentration of about 0.001 (OD)_600nm_. The bacterial culture was incubated at 24 °C for 96 h to allow the *Xcc* biofilm to form. In order to assess the antibiofilm activity and the influence on cell viability of treatments, the mature biofilms were incubated for 4 h and treated as follows: (1) with 6PP (0.001 μg/mL); (2) without 6PP; (3) with the phage alone (10^8^ PFU/mL); (4) with the complex of phage (10^8^ PFU/mL) plus HA (10 mg/mL) and 6PP (0.001 μg/mL). The lowest concentration of 6PP showing the maximum efficacy in combination was selected for the experiments. The biofilm cell and the microscopic observations were performed as previously reported [21].

### 2.6. CLSM Analysis for Dynamic Biofilm Evaluation

The analysis of *Xcc* biofilms was performed using a three-channel flow cell chamber (IBI Scientific, Peosta, IA). A solution of phosphate-buffered saline (PBS, pH 7) was introduced into each channel of the cell at a controlled flow rate of 160 μL/min using an Ismatec IPC 4 peristaltic pump (Cole-Parmer GmbH, Germany). The flow system was kept free of air bubbles using a bubble trap, which created low positive pressure under the PBS flow. Then, a bacterial suspension of *Xcc* at optical density of 0.5 mL^−1^ was left in circulation through the system for 2 h, and the non-adhering cells were removed using sterile PBS for 15 min. Finally, fresh medium (nutrient broth 50% *v*/*v* in PBS) was put through the system for 48 h to allow biofilm formation. After incubation, treatments of fresh medium (NT), 6PP (0.001 μg/mL) or the φHA6PP complex was circulated separately for 3 h into each cell channel. The biofilm formation was evaluated by CLSM. The biofilm cell viability and microscopic observations were determined as previously reported [22].

### 2.7. RNA Extraction and Expression Profiling by qPCR

Fifty milliliters of *Xcc* was incubated at 24 °C for 72 h in a narrow-mouth glass Erlenmeyer flask under static conditions. Then, the phage (10^8^ PFU/mL), 6PP (0.001 μg/mL) and φHA6PP were added to each sample. Ten milliliters of biofilm were collected after 30, 60, 90 and 120 min. Total RNA was extracted using the TRIzol protocol [23]. A NanoDrop ND-1000 (Thermo Fisher Scientific Inc.) was used to assess total RNA quantity. One microgram of purified total RNA was used as a template for first-strand cDNA synthesis using SuperScript III Reverse Transcriptase (Invitrogen). The primers were designed using the tool provided at https://www.eurofinsgenomics.eu/en/ecom/tools/qpcr-assay-design/ for all genes (Supplementary Table). Gene transcript levels were measured using Power SYBR Green PCR Master Mix as previously reported [24]. Thermocycler conditions were as follows: initial step at 95 °C for 10 min, 40 cycles of 95 °C for 15 s, (*clp* 57.1 °C; *manA* 55 °C, *rpf* 59.9 °C, *gumB* 63.7 °C) for 40 s and 72 °C for 1 min. All samples were normalized to HcrC as the reference housekeeping gene. The relative quantitative expression was determined using the 2^−ΔΔCT^ method [25].

## 3. Results

### 3.1. Antibiofilm Activity of 6-Pentyl-α-pyrone

**Figure 1:**
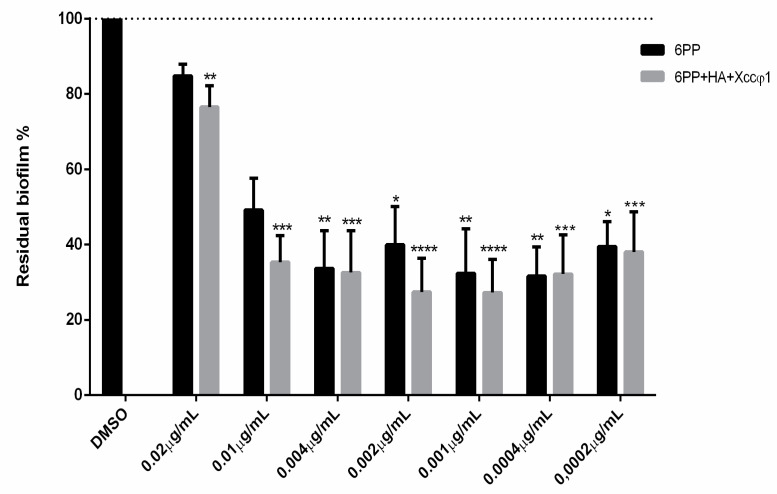
Antibiofilm activities of different concentrations of 6-pentyl-α-pyrone (6PP) alone, and in the complex of 6PP+hydroxyapatite and *Xanthomonas campestris* pv. *campestris* phage (Xccφ1). Biofilm was assessed after 72 h of incubation at 25 °C using a crystal violet assay. The data are expressed as percentages of residual biofilm. Values represent the mean ± SD of the three independent experiments. Absorbance, compared to the untreated control, was considered statistically significant with *p* < 0.05 (* *p* < 0.05, ** *p* < 0.01, *** *p* < 0.001, **** *p* < 0.0001) according to two-way ANOVA multiple comparisons.

The antibiofilm activity of 6PP alone and the complex φHA6PP were evaluated against *Xcc*. The complex φHA6PP limited the biofilm formation more efficiently than 6PP used alone. The 6PP at a concentration of 0.001 μg/mL disrupted the biofilm by about 70%. The DMSO (used as solvent of 6PP at 0.1% concentration) did not interfere with biofilm integrity (Figure 1).

### 3.2. CLSM Analysis for Static Biofilm Evaluation

**Figure 2:**
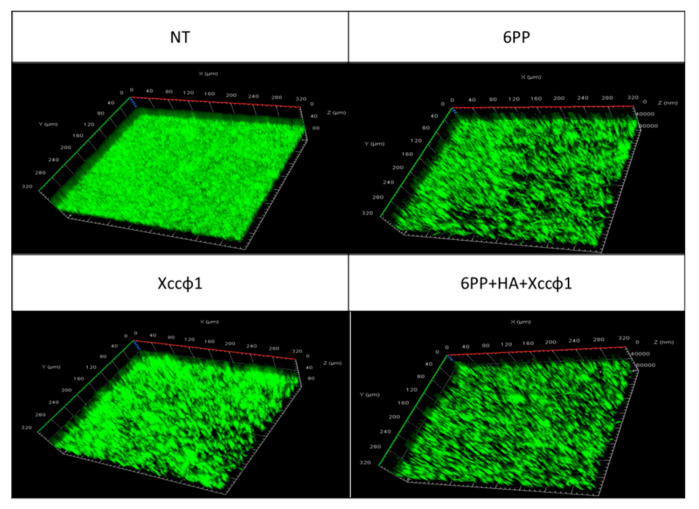
Confocal laser scanning microscopy (CLSM) observation of biofilm formation of *Xanthomonas campestris* pv. *campestris* under static conditions. The treatments were fresh medium, no treatment (NT); 6-pentyl-α-pyrone (6PP, 0.001 μg/mL); *Xanthomonas campestris* pv. *campestris* phage (Xccφ1); and the complex of 6PP, hydroxyapatite (HA) plus Xccφ1. Biofilm analysis was carried out on mature biofilm after 72 h of incubation at 24 °C. The three-dimensional biofilm structure was demonstrated using the LIVE/DEAD Biofilm Viability kit.

CLSM analysis confirmed the results obtained from the crystal violet test. As shown in Figure 2, the 6PP (0.001 μg/mL) and the phage, when used alone, caused a small reduction in the biofilm mass. However, when 6PP was combined with phage and HA, there was a consistent reduction in biofilm formation.

### 3.3. CLSM Analysis for Dynamic Biofilm Evaluation

**Figure 3:**
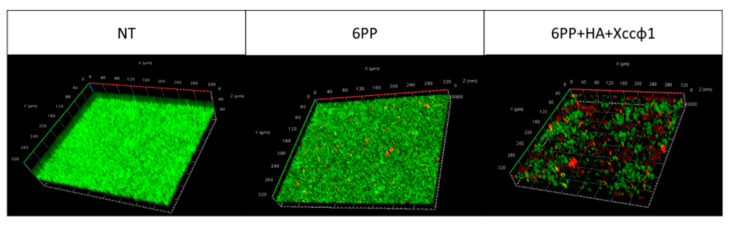
Confocal laser scanning microscopy analysis demonstrating biofilm formation of *Xanthomonas campestris* pv. *campestris* under dynamic conditions. The three treatments were fresh medium–no treatment (NT); 6-pentyl-α-pyrone (6PP, 0.001 μg/mL); and the complex of 6PP, hydroxyapatite (HA) plus *Xanthomonas campestris* pv. *campestris* phage (Xccφ1). Biofilm formation was performed using a three-channel flow cell. The three-dimensional biofilm structures were obtained using the LIVE/DEAD Biofilm Viability kit.

In order to reproduce the plant xylem, the *Xcc* biofilm formation was investigated using a three-channel flow cell system. The phage alone did not show significant disruption of the biofilm structure. The 6PP (0.001 μg/mL) used alone partially disrupted the biofilm structure. However, the biofilm mass was almost totally unstructured when 6PP was combined with the phage and HA (Figure 3). The CLSM fluidic system confirmed the antibiofilm activity of φHA6PP.

### 3.4. RNA Extraction and Expression Profiling by qPCR

**Figure 4:**
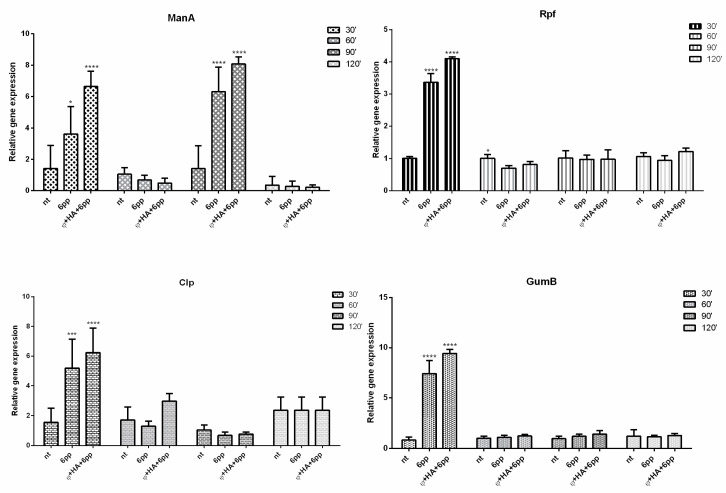
The expression profiles of the four genes (*rpf* at top right; *gumB* at bottom right; *clp* at bottom left; *manA* at top left) of *Xanthomonas campestris* pv. *campestris* by quantitative real-time PCR (qPCR). Biofilm samples untreated (NT), treated with 6-pentyl-α-pyrone (6PP); and the complex of hydroxyapatite, 6PP and *Xanthomonas campestris* pv. *campestris* phage (φHA6PP) were analyzed after 30, 60, 90 and 120 min. Statistical analysis was performed using two-way ANOVA multiple comparison test (* *p* < 0.05, ** *p* < 0.01, *** *p* < 0.001, **** *p* < 0.0001). The gene *HcrC* was used as reference.

The expression levels were evaluated of four principal genes (*rpf*, *gumB*, *clp* and *manA*) involved in the biofilm production pathways. The gene *rpf* positively regulates the synthesis of extracellular enzymes and extracellular polymeric substance (EPS) as well as several essential factors of pathogenicity and diffusible signal factor (DSF) [26]. The gene *clp* is essential in DSF production, and it is highly conserved in *Xcc* [27]. The gene *gumB* is involved in the assemblage of pentasaccharides, transfer of nonglycosidic constituents and export of xanthan [26]. Finally, the gene *manA* encodes several mannase enzymes, including mannan endo-1,4-β-mannosidase, which is a dispersing biofilm enzyme of *Xcc*. Analysis by qPCR showed a significant upregulation of *rpf*, *gumB* and *clp* genes 30 min after treatment, and the complex φHA6PP induced the highest level of upregulation (*p* < 0.05). Interestingly, the upregulation of *manA* was cyclical (every 30 min) (Figure 4).

## 4. Discussion

Worldwide research on bacterial biofilms is proceeding on several diverse fronts, with particular emphasis on the investigation of specifically expressed genes, the role of biofilms in antimicrobial resistance, the evaluation of control measures and the development of innovative strategies.

Many molecules have antibiofilm properties as well as antimicrobial activities [28]. Specifically, we investigated the biofilm formation during *Xcc* growth that plays a key role in its infective process [27]. Our results suggested that 6PP did not show as much significant antibacterial activity against free-living *Xcc* as other bacteria in the biofilm matrix (data not shown). However, this molecule displayed an effective biofilm-dissolving ability. Herein, we are reporting for the first time on the antibiofilm activity of 6PP, a secondary metabolite isolated from the culture filtrate of *T. atroviride* P1. Several species belonging to *Trichoderma* are widely known and recognized as biocontrol and biostimulant agents [29,30,31].

Additionally, the mineral HA is known to chemically interact not only with molecules but also with biological structures like bacteriophages [16]. In fact, the low degree of crystallinity and the presence of carbonate ions in the crystal structure make HA extremely reactive in biological systems and suitable for interaction with, and transportation of, bacteriophages. In this study, HA seems to work well as a carrier for 6PP and Xccφ1 in order to increase their activities against the pathogenic bacteria.

The compound 6PP, alone or in combination with Xccφ1 and HA, showed lower antibiofilm activity when applied at higher concentration (Figure 1, 0.02 mg/mL). It is likely that higher doses of 6PP may interfere with the bacterial endosmosis processes of damaging the cell membranes [32]. Otherwise, it is possibly the formation of aggregates that makes the compound unable to penetrate the biofilm structure [33].

Generally, the complex φHA6PP was more efficient on biofilm under the dynamic rather than under the static conditions. This combination showed potentially valid properties for further in vivo tests.

The DSF biosynthesis is modulated by a novel post-translational mechanism involving protein–protein interaction between the two DSF clusters, synthase RpfF (*rpf*) and sensor RpfC (*gumB*) [34]. Further, the quorum sensing (QS) signal is coupled with several intracellular regulatory networks through the second messenger cyclic dimeric GMP and the global regulator *clp* (gene investigated). Genomic analysis shows that the DSF-QS signaling pathway regulates diverse biological functions including virulence, biofilm dispersal and ecological competence. Future investigations could be made into the DSF-QS system in plant and human bacterial pathogens [35].

The RpfC/RpfG two-component system plays a key role in DSF signal transduction and modulates downstream DSF regulon by changing intracellular content of cyclic dimeric GMP. The increase in the content of cyclic dimeric GMP can positively influence both *manA*, involved in biofilm dispersal, and *clp*, associated with xanthan production by upregulating the gum genes (*gumB*) [34]. Figure 4 shows that, 30 min after φHA6PP treatment, all the genes are upregulated (*rpf*, *clp*, *gumB* and *manA*); this may be related to the promoter *rpf*. Interestingly, after 90 min, only *manA* is upregulated, in accordance to recent work demonstrating that the one-step growth curve of the phage completes its lytic cycle over the exact same period of time [35]. Moreover, *manA* is involved in mannose metabolism, for which the metabolic alteration activity of the phage on *Xcc* biofilm has been previously demonstrated [36]. Specifically, 30 min after treatment with the complex a stress signal, released from the bacteria activates all the pathways (studied) involved in biofilm formation. Subsequently, the activity of the phage, improved by HA and 6PP, may influence the *manA* synthesis in order to assist the biofilm dispersion. This hypothesis is supported by our results in which the treatment with the phage shows high levels of galactomannan that reduce the viscosity of the biofilm. This study highlights the ability of the complex to dysregulate the metabolic pathways via *manA*, a gene involved in processes able to promote the mature biofilm dispersion, thus making the bacterial aggregate more penetrable. For these reasons, the φHA6PP complex seems to work as a DSF molecule, able to activate *manA* expression. Furthermore, the interference on metabolic pathways involved in biofilm formation due to Xccφ1, alone or in combination with HA and eicosanoic acid, has been demonstrated [36]. Our results have provided evidence that the φHA6PP complex modifies the biofilm structure and production, thus probably interacts by biofilm solubilisation.

In conclusion, this study demonstrates that the φHX6PP complex interferes with biofilm production and promotes the mature biofilm dispersion of *Xcc*. This approach may represent a novel strategy for biofilm prevention and control of plant pathogenic bacteria.
